# Anhydrous reverse micelle nanoparticles: new strategy to overcome sedimentation instability of peptide-containing pressurized metered-dose inhalers

**DOI:** 10.1080/10717544.2016.1269850

**Published:** 2017-02-09

**Authors:** Zhengwei Huang, Han Wu, Beibei Yang, Longkai Chen, Ying Huang, Guilan Quan, Chune Zhu, Xing Li, Xin Pan, Chuanbin Wu

**Affiliations:** 1 School of Pharmaceutical Sciences, Sun Yat-Sen University, Guangzhou, Guangdong, P.R. China and; 2 School of Pharmaceutical Sciences, School Southern Medical University, Guangzhou, Guangdong, P.R. China

**Keywords:** Pressurized metered dose inhaler, anhydrous reverse micelle nanoparticle, sedimentation instability, steric barrier effect, homogenous particle size

## Abstract

The objective of this study was to develop a novel anhydrous reverse micelle nanoparticles (ARM-NPs) system to overcome the sedimentation instability of peptide-containing pressurized metered-dose inhalers (pMDIs). A bottom-up method was utilized to fabricate ARM-NPs. Tertiary butyl alcohol (TBA)/water system, freeze-drying and lipid inversion method were successively used to produce the ARM-NPs for pMDI. Various characteristics of ARM-NPs were investigated including particle size, morphology, secondary structure of the peptide drug, aerosolization properties and storage stability. As revealed by the results, ARM-NPs with spherical shape possessed 147.7 ± 2.0 nm of particle size with 0.152 ± 0.021 PdI. The ARM-NPs for pMDI had satisfactory fine particle fraction (FPF) value of 46.99 ± 1.33%, while the secondary structure of the peptide drug was unchanged. Stability tests showed no pronounced sedimentation instability for over 12 weeks at 4–6 °C. Furthermore, a hypothesis was raised to explain the formation mechanism of ARM-NPs, which was verified by the differential scanning calorimetry analysis. The lecithin employed in the reverse micelle vesicles could serve as a steric barrier between peptide drugs and bulk propellant, which prevented the instability of peptide drugs in hydrophobic environment. Homogenous particle size could avoid Ostwald ripening phenomenon of particles in pMDIs. It was concluded that the ARM-NPs for pMDI could successfully overcome sedimentation instability by the steric barrier effect and homogeneous particle size.

## Introduction

The peptide drugs with instable nature are liable to hydrolysis in aqueous environment (Leeb et al., 2015) and enzymolysis in the gastrointestinal tract (Kirby, 2000), making it scarcely feasible for oral administration. Hence, injections are commonly employed in peptide drugs delivery (Antosova et al., 2009). Nevertheless, as an invasive administration route for those peptide drugs which should be applied repeatedly, injection will cause pain, infection and ultimately poor compliance in patients. Injection needs to be performed by the professionally trained medical staff, leading to high inconvenience. These issues have critically limited further development and application of peptide drugs. Therefore, it is urgent to explore new strategies for peptide drug delivery systems.

The pulmonary drug delivery system (PDDS) is a promising peptide drug delivery system, which can provide a comparable bioavailability against injection due to the huge absorptive area and extensive vasculature in lung (Stone et al., 1992; Lombry et al., 2002; Kaur et al., 2016). Low enzyme activity is observed in the lower respiratory tract (Ibrahim & Garcia-Contreras, 2013), favoring the stability of peptide drugs. Thus, PDDS is considered to be an ideal administration route for peptide drugs, which retains the virtue and meanwhile overcomes the shortcomings of injection.

Stability is the most important factor, which should be taken into consideration in peptide-containing PDDS design (Shoyele & Slowey, 2006). Peptide drugs will go through denaturation process if their stability is unmet. The chemical and physical stability of the peptides in PDDS will be different in the nebulizer, dry powder inhaler and pressurized metered-dose inhaler (pMDI), the three approaches of PDDS (Weber et al., 2014; Chen et al., 2016). In pMDI, the propellant-based anhydrous and aseptic concealed environment is capable of avoiding hydrolysis and microbial decomposition of the peptide drugs (Bailey & Berkland, 2009), and the chemical stability of pMDI can be ensured.

However, sedimentation instability is most likely to take place in peptide-containing pMDI. The peptide drugs are supposed to suspend rather than dissolve in the pMDI because of their low solubility in the propellant, resulting in a dynamic driving force toward sedimentation. Direct contact of peptide drugs and propellant will lead to the destruction of hydrated layer and accompanying sedimentation (Williams & Liu, 1999). The Ostwald ripening phenomenon (Welin-Berger & Bergenstahl, 2000) will occur if the particle sizes of pMDI are not homogeneous. Briefly, those particles with originally higher particle size will continuously enlarge and accelerate sedimentation. It can be anticipated that the safety and efficacy of peptide-containing pMDI will be severely challenged after sedimentation. The pMDI would be a prospective approach of PDDS for peptide drugs only if the sedimentation instability is surmounted.

Two basic demands should be satisfied in order to overcome the sedimentation instability of peptide-containing pMDI: (1) steric barrier between peptide drugs and propellant and (2) homogenous particle size. It is not advisable to attempt to dissolve peptide drugs in propellant because the poor solubility of peptide drugs in propellant will bring in extra sedimentation instability issue. On the basis of such principles, several nanoparticulate systems were established, among which polymeric particulate carrier (Sharma et al., 2012) and microemulsion (Shan et al., 2014) are most intensively studied. In these systems, polymer or emulsifier served as a steric barrier between peptide drugs and propellant, while uniform nano-metered particle sizes assured dynamical stability. Additionally, nanoparticles can escape from macrophage phagocytosis clearance (Zhang et al., 2011), favorable for bioavailability enhancement.

However, additives for pMDI in these studies were mostly not approved by FDA or generally recognized as safe. To develop a safer formulation for peptide-containing pMDI, a novel system based on anhydrous reverse micelle nanoparticles (ARM-NPs) was investigated in this paper. Composed by FDA-approved lecithin (Nyambura et al., 2009), ARM-NPs are an innovative type of nanoparticulate system, which can simultaneously offer steric barrier effect and guarantee homogeneous particle size.

This study was intended to explore the sedimentation stability of peptide-containing ARM-NPs for pMDI and therefore verify the steric barrier effect and homogeneous particle size of ARM-NPs. Salmon calcitonin (sCT) whose major indication is osteoporosis (Huang et al., 2006) was selected as the model peptide drug. It is worth mentioning that the chemical stability of sCT is satisfactory, and a commercial sCT aqueous nasal spray named Fortical® had already been marketed (Touitou & Illum, 2013). Hence, the only instability factor of sCT containing pMDI should be sedimentation instability. A co-solvent system (Cui et al., 2006; Tan et al., 2011,2012), freeze-drying and lipid inversion method were utilized to fabricate ARM-NPs for pMDI. A rational hypothesis about the formation of ARM-NPs was put forward and confirmed by the subsequent thermal analysis. It was expected that such system could successfully overcome sedimentation instability by the steric barrier effect and homogeneous particle size.

## Materials and methods

### Materials

99.5% of purity sCT was used as a model peptide and purchased from Chengdu Kaijie Peptide Co., Ltd. (Chengdu, China). Soy lecithin (Lipoid S100) was purchased from Lipoid GmbH (Ludwigshafen, Germany). Tertiary butyl alcohol (TBA) and ethanol absolute were obtained from Tianjin Guangfu Fine Chemical Research Institute (Tianjin, China). HFA 134a was obtained from INEOS Ltd. (Runcorn, UK). Tetramethylammonium hydroxide (TMAH) was purchased from Aladdin Industrial Inc. (Shanghai, China). Acetonitrile (HPLC grade) was obtained from Burdick & Jackson® (Muskegon, MI). All of the reagents were used as received without further purification. A water purification system PureLAB option (ELGA Lab Water Inc., High Wycombe, United Kingdom) was utilized to provide super purified water.

### Methods

#### Optimization of sCT-containing ARM-NPs formulation

The TBA volume was controlled below 250 μL to ensure the formation of double-layer original lipid vesicles according to the ternary phase diagram studies (data not shown). It was borne in mind that the amount of TBA and lecithin applied in the system would influence the particle size and morphology of original lipid vesicles and ARM-NPs. Therefore, different volumes of TBA (i.e. 150, 200 and 250 μL) and different concentrations of lecithin (i.e. 5%, 10% and 20%) in the organic phase were scanned in the formulation optimization. Totally, there were nine investigated formulations whose TBA volumes and lecithin concentrations are summarized in Table 1.

**Table 1.** The TBA volume and lecithin concentration in each formulation and the detected particle size and PdI of the obtained original lipid vesicles.

Formulation	TBA volume (μL)	Lecithin concentration (%, w/v)	Particle size (nm)	PdI
1	150	5	174.8	0.290
2	200	5	161.9	0.202
3	250	5	145.5	0.084
4	150	10	241.9	0.360
5	200	10	176.5	0.244
6	250	10	166.0	0.138
7	150	20	266.2	0.375
8	200	20	206.1	0.267
9	250	20	177.9	0.264

A bottom-up method was adopted to fabricate the ARM-NPs. The organic phase was prepared by completely dissolving pre-determined mass of lecithin into TBA, while the aqueous phase was obtained by dissolving 2 mg of sCT and 1 mg trehalose (cryoprotectant) into 1 mL super purified water. The organic phase was added dropwise into the aqueous phase under 37 °C water bath and stirring at a rate of 1300 rpm, producing sCT containing original lipid vesicles. With the purpose of removing impurities and microorganism, the system was filtered through a 0.22-μm nylon-66 membrane (Membrana GmbH, Wuppertal, Germany). The filtrate was instantly snap frozen by being immersed in liquid nitrogen and thereafter freeze-dried by CHRIST ALPHA 1-4LSC freeze dryer (Osterode, Germany) under a condition of −50 °C and 0.25 mbar for at least 12 h to remove both water and TBA thoroughly. Ethanol absolute was added into the obtained lyophilized complexes to yield ARM-NPs by lipid inversion. Finally, formulation was optimized by analyzing the results of particle size measurements via one-way ANOVA and estimated marginal means using SPSS 19.0 software (IBM Corporation, Armonk, NY). The content of sCT in optimal formulation was quantified chromatographically.

In addition, it was well documented that shear force (stirring rate) during preparation had direct impact on particle size (Nyambura et al., 2009). Intended to screen the optimal stirring rate, different stirring rates (430, 870 and 1300 rpm, respectively) when mixing the aqueous phase and organic phase was examined. Three samples for each stirring rate were prepared. The other procedures were as same as described above.

#### Dynamic laser scattering analysis for ARM-NPs

The size distribution characteristic of original lipid vesicles, ARM-NPs and reconstructed liposomes, which obtained by exposing the ARM-NPs to ample volume of water, was measured *via* dynamic laser scattering (DLS) method by Malvern Zetasizer Nano ZS90 (Malvern Instruments, Malvern, UK). Two main parameters, namely hydrodynamic diameter which was expressed as particle size and polydispersity index (PdI) indicating the width of particle size distribution, were determined. Initially, by sonication (Ningbo Xinzhi Biotechnology Co. Ltd., Zhejiang, China) in a water bath for 5 min, the samples were dispersed uniformly in isooctane, which was previously filtered through a 0.1-μm nylon-66 membrane (MEMBRANA®). Since it was necessary to provide required analytical count rate of no less than 50 kilo counts per second in DLS, the concentration of sample suspensions was adjusted to 5 mg of sample/1 mL of isooctane. Suspensions were successively transferred into a non-frosted quartz cuvette, which was allowed to stand for 2 min prior to measurement for equilibrium. Each sample was analyzed in triplicate.

#### Transmission electron microscopy (TEM) studies

Suspended in isooctane, trace amount of the original lipid vesicles, ARM-NPs and reconstructed liposomes was placed on copper grids to allow evaporation of isooctane. The ARM-NPs were negatively stained with 1% (w/v) phosphotungstic acid solution, and the excessive solution was absorbed. Before being examined by a JEM-1400 transmission electron microscope (JEOL Ltd., Tokyo, Japan), the samples were dried under a condition of relative humidity less than 45% at room temperature.

#### Secondary structure determination of sCT

*Fourier transformation infrared spectroscopy study.* Lyophilized complexes obtained during the ARM-NPs preparation process, atomized sCT from pMDI formulation, pure sCT and freeze-dried free sCT, which employed the same process parameters as sCT containing ARM-NPs were collected for further study. Appropriate amount of these samples were carefully mixed with potassium bromide (KBr) powder (spectroscopy grade, Sinopharm Chemical Reagent Co., Ltd, Shanghai, China) and compressed into tablets for examination by a KBr beam splitter (TEN-SOR37, Bruker Corporation, Bremen, Germany) under a pressure of 10 tons for 60 s. Samples were scanned by transmission mode within the range of 400–4000 cm^−1^, with a resolution of 4 cm^−1^. 1600–1700 cm^−1^ or the amide bands of sCT in each sample were compared in detail. Also, deconvolution of 1600–1700 cm^−1^ bands and Gauss fitting were conducted by the software Bruker OPUS 6.5 for analysis.

*Circular dichroism spectroscopy analysis*. Identical weight of the obtained lyophilized complexes, atomized sCT from pMDI formulation, pure sCT and pure sCT added with appropriate amount of protein denaturing agent sodium dodecyl sulfate (SDS) were respectively dissolved in super purified water to a concentration of 100 μg/ml (sCT). Circular dichroism (CD) spectra were recorded on a Jasco J-810 spectropolarimeter (Chirascan type, Applied Photophysics Ltd., Surrey, UK) equipped with JASCO software. The instrument parameters were adjusted to 190–250 nm wavelength, 1 nm bandwidth, 2 s response time and 25 °C with an average of three scans.

#### Preparation of ARM-NPs for pMDI

Two-step filling method for pMDI described in detail elsewhere (Tan et al., 2012) was also utilized here. One millilieter of sCT containing lipid vesicles suspension was prepared and freeze-dried following the procedure described above. The lyophilized complex was moistened with 1 mL ethanol absolute by ultrasonic treatment for 1 min to produce ARM-NPs, which were later transferred into a plastic-coated glass bottle (Shandong Jewim Pharmaceutical Co. Ltd., Shandong, China). Ten grams of HFA 134a as the propellant was perfused into the bottle through a reliable valve system by an aerosol filling machine (Zhongshan Zhihua Aerosol Equipment Co. Ltd., Guangdong, China) at controlled conditions (22–26 °C and 40–50% relative humidity). Ultimately, the obtained pMDIs were sonicated for 2 min to ensure homogeneity.

#### Aerosolization properties of pMDIs

In order to explore their aerosolization properties, the pMDIs were applied to twin stage impinger (TSI, National Center for Pharmaceutical Engineering, Shanghai, China) in accordance with British Pharmacopeia 2015. The upper and lower stages were loaded with pH 7.0 PBS (7 and 30 mL, respectively), and the air flowing rate was adjusted to 60 L/min by a glass rotameter (Model LZB-10, Changzhou Chengfeng Flow Meter Co., Ltd. Jiangsu, China). 0.3 mm orifice actuators (Valois (Suzhou) Dispensing Systems Co., Ltd, China) were utilized. The pMDIs were primed by actuating five shots to waste prior to the tests. For each pMDI, 10 actuations were sprayed into the TSI, and approximate 10-s breaks were set between actuators to avoid cooling effect of HFA 134a. After each discharge, the air pump was allowed to run for 5 s. The air pump was switched off for another 5 s and meanwhile the inhaler was shaken manually. TSI was dismantled and sCT deposited in every region (Actuator, Throat, Stage 1 and Stage 2) was dissolved in appropriate volume of pH 7.0 PBS for quantification by HPLC after each run. Each aerosolization test was conducted in triplicate. For analysis, recovered dose (RD) and fine particle dose (FPD) were defined as total mass of sCT detected in TSI (Actuator + Throat + Stage 1 + Stage 2) and mass of sCT determined in Stage 2 solely which had an effective cutoff diameter of 6.4 μm, respectively. Further, fine particle fraction (FPF) was calculated as the ratio of FPD/RD multiplied by 100%.

#### Investigation of storage stability of pMDIs

A batch of pMDIs was stored in different controlled conditions (i.e. 4, 20 and 30 °C). Dynamic laser scattering analysis was conducted on day 0, 5 and 10 to characterize the particle size distribution of pMDIs during storage. Storage stability of pMDIs with valve-up was investigated for 1, 6 and 12 weeks. The sCT containing pMDIs were placed in a plastic container at 4–6 °C, while aerosolization properties were tested by MSI and the content of drug per press was determined by dosage unit sampling apparatus for MDIs (DUSA) in accordance with European Pharmacopeia 8.0 at corresponding time intervals. The 1-min stability was evaluated visually by manually shaking the pMDIs to investigate the redispersibility of the formulation. This operation was realized in triplicate.

#### Differential scanning calorimetry analysis

The phase diagram of TBA/water system had been well studied by Kasra Kasraian (1995), which illustrated that the frozen crystallization behavior of TBA/water system would vary with TBA/water ratio (v/v). For further investigation, differential scanning calorimetry (DSC) experiment was conducted to explore the thermodynamic behavior of TBA/water system during freeze-drying. Approximately 5 mg of following samples were tested in DSC: TBA/water 1:4, TBA/water 1:4 + lecithin, TBA/water 3:2, TBA/water 3:2 + lecithin and lipid vesicle suspension. The onset of freezing was measured by NETZSCH DSC 200 F3 Maia (NETZSCH-Gerätebau GmbH, Selb, Germany) with a pre-determined protocol: The samples were successively frozen to −40 °C, heated to −7 °C at a rate of 5 °C/min, maintained thermostatic for 10 min, again frozen to −40 °C and heated to 40 °C at a rate of 5 °C/min.

#### Chromatographic quantification method of sCT

Concentration of sCT in aerosolization tests was determined by HPLC system (Shimadzu LC-20AT liquid chromatograph, Shimadzu Co., Ltd., Kyoto, Japan) equipped with a reverse-phase Phenomenex Jupiter C_18_ column (5 μm, 4.6 × 250 mm, Phenomenex Inc., Torrance, CA) at 40 °C with UV detection at 220 nm. Acetonitrile (mobile phase A)–0.02 mol/L TMAH solution (pH 2.5, mobile phase B) was adopted as mobile phase at 1.000 L/min. A program of dual gradient elution was run as *T* (min)/%mobile phase A: 0–15 min, 72%; 15–17 min, 63%; 17–30 min, 72%. Injection volume was adjusted to 100 μL. A calibration curve was constructed by sCT standard solutions of 0.8–50 μg/mL (*R*^2 ^=^ ^0.9997). Each sample was quantified in triplicate.

#### Statistical analysis

Data acquired were expressed as mean ± SD and analyzed using one-way ANOVA followed by Dunnett’s post hoc by SPSS 19.0 software if necessary. In all cases, *p* values less than 0.05 were considered statistically significant.

## Results

### Optimization of sCT containing ARM-NPs formulation

Organic phase (lecithin dissolved TBA) was added dropwise into aqueous phase (sCT with trehalose) to produce original lipid vesicles. Volume of TBA and concentration of lecithin were optimized during the preparation process in order to obtained original lipid vesicles within proper size range. Particle size and PdI of original lipid vesicles were measured and listed in Table 1.

The increase of TBA volume from 150 to 250 μL led to the reduction of both particle size and PdI with a certain lecithin concentration, demonstrating that the particle size and the width of particle size distribution for original lipid vesicles decreased as the TBA volume increased. It had been reported that excess TBA might destroy the structure of lipid vesicles (Cui et al., 2006), resulting in drug leakage and low reproducibility. Hence, TBA volume higher than 250 μL was omitted in this study. Increased lecithin concentration would raise the particle size and PdI with fixed TBA volume, as shown by the current results. It should be noted that insufficient amount of lecithin could not effectively form the lipid vesicles. Thus, the lecithin concentration below 5% was not explored.

In order to determine an optimized formulation, one-way ANOVA and estimated marginal means analyses were conducted (Table 2).

**Table 2.** Results of estimated marginal means of the particle size and PdI.

					95% Confidence interval	
Parameter	Formulation compositionc	Mean	Std. error	Lower bound	Upper bound	
Particle size	TBA volume (μL)					
	150	227.6	10.2	199.2	256.1	
	200	181.5	10.2	153.1	209.9	
	250	163.1	10.2	134.7	191.6	
	Lecithin concentration (%)					
	5	160.7	10.2	132.3	189.2	
	10	194.8	10.2	166.4	223.2	
	20	216.7	10.2	188.3	245.1	
PdI	TBA volume (μL)					
	150	0.342	0.021	0.285	0.399	
	200	0.239	0.021	0.182	0.296	
	250	0.162	0.021	0.105	0.219	
	Lecithin concentration (%)					
	5	0.192	0.021	0.135	0.249	
	10	0.248	0.021	0.191	0.305	
	20	0.303	0.021	0.246	0.360	

Table 2 reveals that least estimated marginal means could be achieved when TBA volume was given its maximum (250 μL) and lecithin concentration was given its minimum (5%). Combined with the results of Table 1, the optimal formulation was determined and adopted in further studies: Volume of TBA was 250 μL (i.e. TBA/water 1:4, v/v), while concentration of lecithin was 5%. Concentration of sCT in optimal formulation was 170.11 ± 3.41 μg/mL, chromatographically determined.

**Figure 1.**
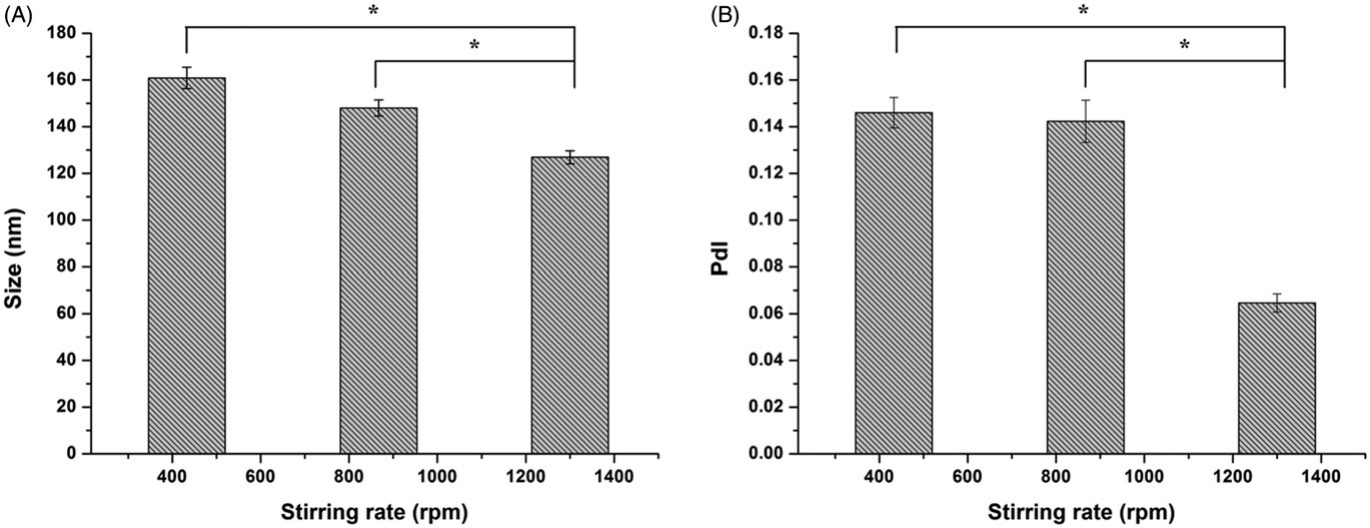
Effect of stirring rate on the vesicle size (A) and PdI (B) of original lipid vesicles. Data represent mean (± SD) of three preparations, *p <* *0.05 with each group.

The stirring rate during preparation process was also optimized in this study. Particle size and PdI were determined and shown in Figure 1. There was no significant difference (both *p *>* *0.05) in particle size and PdI for original lipid vesicles with the stirring rate ranging from 430 to 870 rpm. However, with stirring rate increasing from 870 to 1300 rpm, particle size and PdI significantly decreased to 126.9 ± 2.8 nm (*p *<* *0.05) and 0.065 ± 0.004 (*p *<* *0.05), respectively, implying that there might be a threshold above which stirring rate would have effect on the particle size distribution of the original lipid vesicles. But stirring rate higher than 1300 rpm meant higher energy consumption and possibility for excessive heat generation. Therefore, stirring rate of 1300 rpm was employed in subsequent investigation.

### Morphology of sCT-containing ARM-NPs

**Figure 2.**
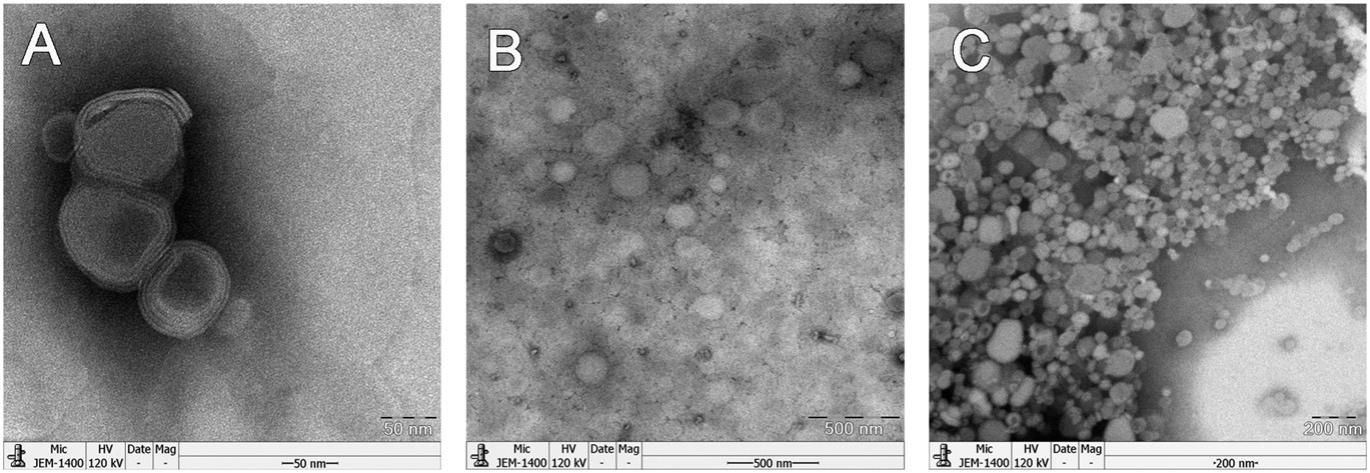
TEM micrographs of the samples: (A) Original lipid vesicles; (B) ARM-NPs; (C) reconstructed liposomes.

TEM micrographs (Figure 2) revealed that the original lipid vesicles, ARM-NPs and reconstructed liposomes were all spherical with ca. 150 nm particle size, among which original lipid vesicles possessed a double-layered structured. The lipid vesicles could act as steric barrier for sCT. Size distribution measured by DLS is summarized in Table 3, and the results of DLS were well in agreement with TEM micrographs. The particle sizes of all samples are less than 200 nm with a PdI less than 0.2, indicating a homogenous particle size distribution in nano-scale (Bhandari & Kaur, 2013). Particle size of ARM-NPs showed no significant difference with original lipid vesicles (*p *>* *0.05) but was significantly smaller than reconstructed liposomes (*p *<* *0.05). It might be owing to the rearrangement of the lipid structure into liposomes with lager volume after exposed to water (Kirby, 2000). There were no statistical significance between PdI of ARM-NPs, original lipid vesicles and reconstructed liposomes (*p *>* *0.05).

**Table 3.** The particle size and PdI of original lipid vesicles, ARM-NPs and reconstructed liposomes.

Sample	Particle size (nm)	PdI
Original lipid vesicles	151.1 ± 1.0	0.119 ± 0.013
ARM-NPs	147.7 ± 2.0	0.152 ± 0.021
Reconstructed liposomes	181.0 ± 4.9	0.102 ± 0.029

### Impact of preparation and atomization process on the secondary structure of sCT

#### FTIR spectroscopy results

**Figure 3.**
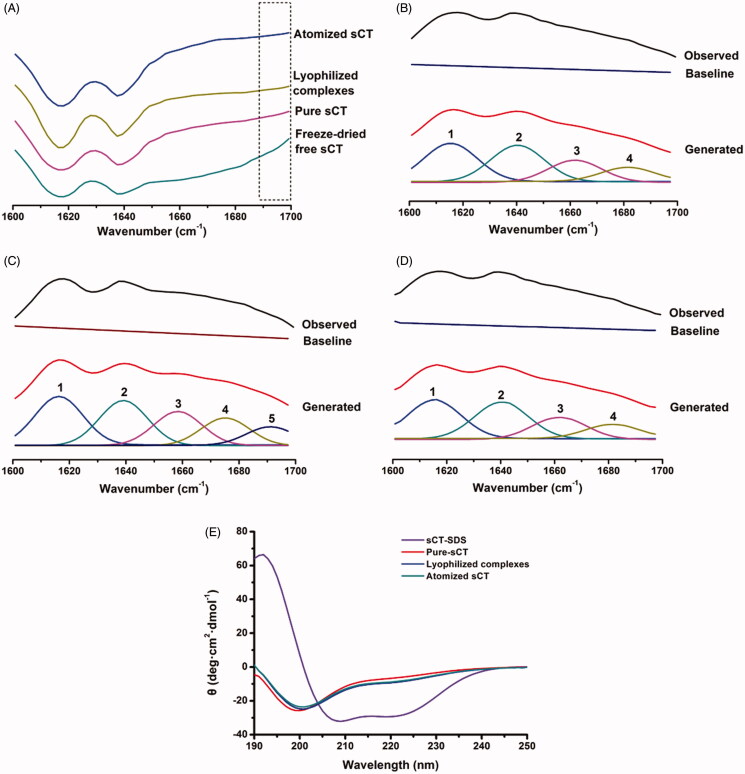
FT-IR and CD results. (A) Comparison of FT-IR spectra: lyophilized complexes, pure sCT, atomized sCT from pMDIs and freeze-dried free sCT; (B) Fitting curves of sCT amide I band of pure sCT; (C) Fitting curves of sCT amide I band of freeze-dried free sCT; (D) Fitting curves of sCT amide I band of atomized sCT from pMDIs; (E) Comparison of sCT CD spectra under different conditions: aqueous solutions of SCT with SDS, pure sCT, atomized sCT from pMDIs and the lyophilized complexes.

The samples were scanned through 400–4000 cm^−1^ under pre-determined conditions by FITR. Specifically, the band within the range of 1600–1700 cm^−1^ could be attributed to the characteristic amide vibration band I of sCT (Lee et al., 2010; Lee & Lin, 2011) and the intercepted spectra are summarized in Figure 3(A). The free-dried free sCT sample possessed different trend from atomized sCT from pMDI, pure sCT and the lyophilized complexes in 1690–1700 cm^−1^ band. Deconvolution and Gauss fitting were performed further to give an unambiguous comparison between the spectra. The curve-fitted spectra of pure sCT, lyophilized free sCT and atomized sCT from pMDI are shown in Figure 3(B), (C) and (D).

Referring to previous studies (Souillac et al., 2002; Lee et al., 2010; Lee & Lin, 2011), the 1–4 bands in Figure 3(B), (C) and (D) could be, respectively, attributed to the β-sheet (1620–1640 cm ^−1^), random coil (1643–1649 cm^−1^), α-helix (1650–1660 cm^−1^) and β-corner (1660–1695 cm^−1^), while the band 5 (1690 cm ^−1^) emerged in Figure 3(C) could be explained by the dehydration of sCT during free-drying and which caused its secondary structure to switch to α-helix. As was well known, dehydration of protein should be carefully avoid so as to prevent denaturation. Figure 3(B) depicts that there were no characteristic peaks near 1690 cm^−1^, demonstrating that the additive trehalose was capable of preventing sCT from structural change throughout the freeze-drying process. This result suggested that trehalose was a qualified cryoprotectant. Similarly, it is indicated by Figure 3(D) that the secondary structure of sCT was not destructed by atomization process.

#### CD spectroscopy results

The obtained CD spectra of aqueous solutions of SCT with SDS, pure sCT, atomized sCT from pMDI formulation and the lyophilized complexes are displayed in Figure 3(E). It has been well documented that the common regularities of CD spectra of peptides were that α-helix showed a positive characteristic peak near 192 nm and two shoulder peaks at 208 and 222 nm, β-sheet exhibited a negative band near 216 nm and β-corner possessed a positive band at 206 nm (Fort et al., 2002; Greenfield, 2006; Creasey et al., 2012; Hollosi et al., 2012).

There was a pronounced α-helix band in sCT-SDS but no corresponding band in pure sCT, indicating that sCT underwent a structural change after denatured by SDS. In addition, the shoulder peaks at 208 and 222 nm in sCT-SDS were obviously strengthened when compared to pure sCT or lyophilized complexes. Taken together, it could be inferred that denaturation of sCT took place, which was in accompany with the occurrence of α-helix structure. Although the spectra of lyophilized complexes and atomized sCT from pMDI formulation showed a slight shift from pure sCT, the secondary structure of sCT was not considered to alter significantly, since no distinct α-helix structure was recorded. By far, it was proven that trehalose as a cryoprotectant could effectively protect sCT from structural change during freeze-drying process and sCT existed in its nature structure in the lyophilized complexes, and that atomization would not significantly affect the secondary structure of sCT.

### Aerosolization properties of pMDIs

**Figure 4.**
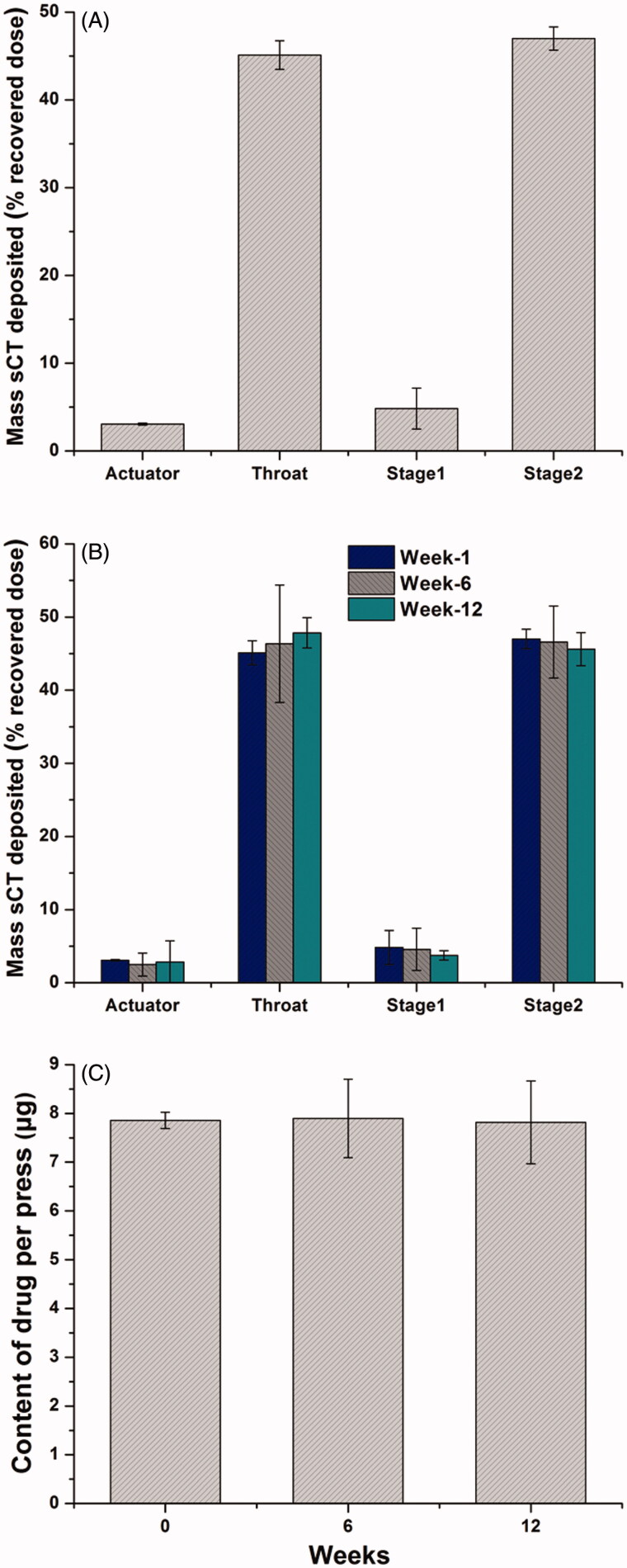
Results of formulation characterization and stability evaluation. (A) The aerosolization properties of the optimized sCT-containing ARM-NPs for pMDIs formulation. Data represent mean (± SD) of three preparations; (B) The aerosolization properties of the optimized sCT-containing ARM-NPs for pMDIs formulation 12-week during storage under 4–6 °C condition. Data represent mean (± SD) of three preparations; (C) Content of drug per press during 12-week storage under 4–6 °C condition. Data represent mean (± SD) of three preparations.

Figure 4(A) depicts the deposition profiles after aerosolization of 10 actuations from the optimized sCT-containing ARM-NPs for pMDIs into the TSI instrument. Generally, the mass fraction of recovered sCT in Stage 2 whose effective cutoff diameter was 6.4 μm was denoted as FPF. FPF value was encouragingly 46.99 ± 1.33%. The low deviation suggested the high reproducibility of the aerosolization behavior of sCT containing pMDIs.

### Storage stability of sCT-containing ARM-NPs for pMDIs

As revealed by Table 4, particle size of pMDIs increased slightly during 10-day storage, but not statistically significant (*p *>* *0.05). PdI of pMDIs markedly increased within 10 days (*p *<* *0.05) while well kept below 0.2. These results suggested that Ostwald ripening was effectively inhibited in the formulations. The pMDI formulations were stored under 4–6 °C condition up to 12 weeks. Both the FPF values (Figure 4B) and content of drug per press (Figure 4C) remained unchanged (*p *>* *0.05) during 12-week storage, indicating that the uniformity and particle size of the pMDIs were well kept with time. Furthermore, the pMDIs could maintain suspension stability for at least 1 min after shaking over 12 weeks, demonstrating that the homogeneity of the formulation could survive the storage process. Taken together, sCT-containing ARM-NPs for pMDI were highly stable during 12-week storage under 4–6 °C condition.

**Table 4.** Characterization of particle size and PdI of sCT containing pMDIs during 10-day storage under different conditions: 4, 20 and 30 °C.

	Particle size (nm)			PdI		
Temperature (°C)	Day 0	Day 5	Day 10	Day 0	Day 5	Day 10
4	160.4 ± 6.6	164.9 ± 5.2	164.2 ± 9.1	0.024 ± 0.002	0.102 ± 0.023	0.171 ± 0.015
20	147.3 ± 0.4	152.9 ± 9.0	150.9 ± 5.5	0.051 ± 0.005	0.178 ± 0.014	0.107 ± 0.011
30	165.1 ± 0.5	163.4 ± 6.0	169.9 ± 9.3	0.065 ± 0.018	0.160 ± 0.028	0.176 ± 0.023

### DSC analysis of TBA/water system

**Figure 5.**
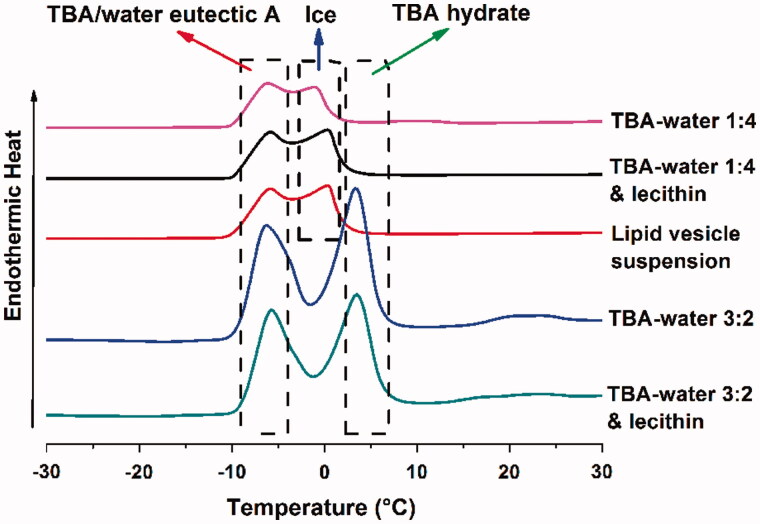
DSC thermograms of TBA/water 1:4, TBA/water 1:4 + lecithin, lipid vesicle suspension, TBA/water 3:2 and TBA/water 3:2 + lecithin.

DSC thermograms of the TBA/water of different ratios and lipid vesicle samples are shown in Figure 5. According to previous study (Kasra Kasraian, 1995), the endothermic peak at approximately 3, 0 and −7 °C could be attributed to the formation of the crystal of TBA hydrate, ice and TBA/water eutectic A, respectively. The addition of lecithin did not alter the position of the endothermic peaks basically, but slightly pushed the TBA hydrate peak rightwards for about 1 °C in TBA/water 1:4 system. TBA/water 1:4, TBA/water 1:4 + lecithin and lipid vesicle suspension (these three were denoted as TBA/water 1:4 system) exhibited the peak of ice and TBA/water eutectic A, while TBA/water 3:2 and TBA/water 3:2 + lecithin (these two were denoted as TBA/water 3:2 system) showed the peak of TBA hydrate and TBA/water eutectic A. Therefore, it was reasonable to believe that TBA/water 1:4 system and TBA/water 3:2 system underwent different frozen crystallization behavior during lyophilization. As for TBA/water 1:4 system, crystal of ice and TBA/water eutectic A generated successively during freeze-drying. As to TBA/water 3:2 system, crystal of TBA hydrate rather than ice would emerge prior to TBA/water eutectic A. As a consequence, the different process might lead to different microscopic structure of the produced nanoparticles, which would be further discussed in the “Discussion” section.

## Discussion

The approach pMDI for peptide drugs delivery has been rapidly gaining momentum since the last decade (Tan et al., 2011,2012; Momoh et al., 2013; Shan et al., 2014). It should be noted that vast majority of peptide drugs are sparsely soluble in the hydrophobic propellant of pMDI. The low dielectric constant of the propellant will induce the peptide drugs with ionic surface residues to aggregate during storage (Affleck et al., 1992). Inhomogeneous particle size is the inducement of sedimentation, according to Ostwald ripening phenomenon (Kirby, 2000). Hence, sedimentation instability will occur and challenge the safety and efficacy of peptide-containing pMDI.

A steric barrier should be constructed between the peptide drugs and the bulk propellant, and meanwhile homogenous particle size should be ensured in order to overcome the sedimentation instability of pMDI. In this study, novel ARM-NPs was investigated as a candidate for such a system. TBA/water system was utilized to fabricate ARM-NPs due to its unique energy-saving advantage in freeze-drying (Kasraian & DeLuca, 1995; Cui et al., 2006). As a bottom-up method, it could avoid the thermal degradation of peptide drugs (Tan et al., 2011,2012). The optimal formulation was determined by exploring several formulation factors: volume of TBA, concentration of lecithin and stirring rate.

As shown by the current results, ARM-NPs with desired particle size, aerosolization properties and stability was fabricated by using 250 μL of TBA, 5% lecithin in TBA (w/v) and 1300 rpm stirring rate. The particle size of ARM-NPs was 147.7 ± 2.0 nm with a PdI of 0.152 ± 0.021 and spherical in shape, demonstrating that the preparation process was able to obtain nanoparticles with a homogeneous nano-metered particle size distribution. It was inferred that the reverse micelle vesicles comprised lecithin could act as steric barrier between sCT and HFA 134a. The steric barrier effect and homogenous particle size were the two major mechanisms for overcoming the sedimentation instability of sCT containing pMDI.

A constant secondary structure was the prerequisite of chemical and physical stability of peptide drugs. Major changes on secondary structure would result in denaturation. As for sCT, the occurrence of α-helix was an indication of denaturation. Denaturation of sCT was avoided by trehalose during freeze-drying process in this study, demonstrated by no marked α-helix bands in Fourier transformation infrared (FTIR) and CD spectra. It was well documented that the stabilization mechanism of trehalose during freeze-drying could be attributed to water replacement (Li et al., 2010). Trehalose was capable of occupying the active sites of hydrogen bonds on the surface of peptide drugs when water was removed during freeze-drying, and accordingly prevented changes of the sCT secondary structure. Also, the secondary structure of sCT did not change during the atomization process where high shear force could be introduced. The unchanged secondary structure made significant contribution to the stability of the ARM-NPs for pMDI.

The aerosolization properties of the ARM-NPs were evaluated, considering that they were designed for pMDI. An FPF value of 46.99 ± 1.33% was obtained in the measurement, indicating that approximately half of the ARM-NPs in pMDI could deposit in the deep lung. Lecithin employed to fabricate ARM-NPs could be classified as non-volatiles. The addition of non-volatiles was reported to be detrimental to FPF value owing to their effect of saturation vapor pressure reduction and viscosity elevation (Selvam et al., 2012). Therefore, the FPF value obtained in this study was much lower than the formulation free of lecithin. For instance, QVAR 80 mg possesses an FPF value up to 70% (Dellamary et al., 2000; Mitchell et al., 2003; Buttini et al., 2014; Doub et al., 2014). By contrast, FPF values determined in those studies applying non-volatiles were basically less than 30% (Selvam et al., 2012; Shan et al., 2014; Ivey et al., 2015). To sum up, the results in present study was quite encouraging.

Evaluations were performed to explore the stability of ARM-NPs for pMDI stemmed from steric barrier effect and homogenous particle size. There was no significant increment of particle size within 10 days at different temperatures, even 30 °C. This demonstrated that Ostwald ripening was effectively inhibited in pMDI formulation. FPF value did not significantly change (*p *>* *0.05) during 12-week storage, suggesting that the ARM-NPs were able to uniformly suspend in HFA 134a without aggregation and maintain a favorable aerosolization diameter for inhalation (Chow et al., 2007; Haughney et al., 2010). Content of drug per press of the pMDI kept at ca. 8 μg during storage process, which confirmed the satisfactory stability of optimized pMDI formulation. In addition, the 1-min stability of the pMDI was well maintained over 12 weeks. This ensured that a homogeneous dispersion could be obtained for inhalation after hand-shaking (de Waard et al., 2008). Taken together, these stability tests had revealed that sCT containing pMDI could maintain highly stable during storage process, which could be attributed to the steric barrier effect and homogenous particle size of ARM-NPs. The steric barrier effect protected sCT from the bulk propellant, while homogenous particle size mitigated the Ostwald ripen phenomenon, concurrently contributing to overcome the sedimentation instability in peptide-containing pMDI.

The formation mechanism of ARM-NPs was investigated. It was worth mentioning that the frozen crystallization behavior varied as a function of composition of the TBA/water system. Kasra Kasraian (1995) established that the phase diagram of the frozen crystallization behaviors of TBA/water system could be categorized into four types (A–D) according to the different mass fractions of TBA in the system (Table 5). The volume ratio of the TBA/water system used in the optimal formulation (1:4) was converted into a mass fraction of 16.32%, which could be assigned to type A. A hypothesis was put forward to demonstrate the formation mechanism of ARM-NPs based on the analysis on the phase diagram and the aforementioned results. For better interpretation, an extra TBA/water system (3:2 v/v or 53.92% w/w) was introduced for comparison.

**Table 5.** The four kinds of frozen crystallization behavior of TBA/water system.

Type	TBA% (w/w)	Phase I (high temperature)	Phase II (moderate temperature)	Phase III (low temperature)
A	0–20	l.s.	Ice + l.s.	Ice + eutectic A
B	20–70	l.s.	TBA hydrate + l.s.	TBA hydrate + eutectic A
C	70–90	l.s.	TBA hydrate + l.s.	TBA hydrate + eutectic B
D	90–100	l.s.	TBA + l.s.	TBA + eutectic B

Note: l.s., liquid solution.

**Figure 6.**
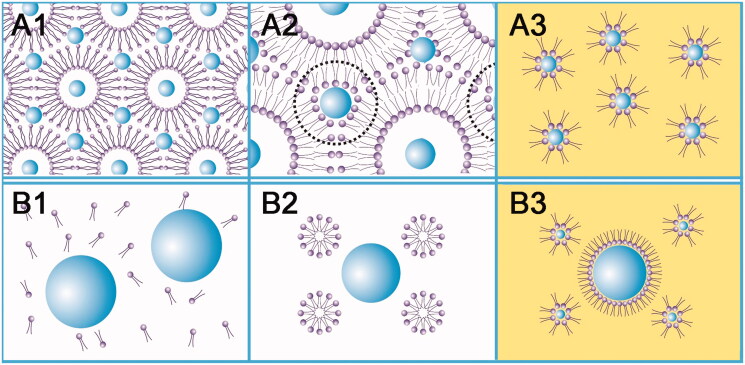
The schematic diagram about the forming process of ARM-NPs. (A) The TBA/water 1:4 system; (B) the TBA/water 3:2 system; (1) before freeze-drying; (2) during freeze-drying; (3) after adding ethanol absolute. Larger balls without tails and smaller balls with tails represented sCT and lecithin, respectively. A1, A2, B1 and B2 referred to aqueous environment while A3 and B3 depicted the addition of ethanol absolute.

The schematic diagram (Figure 6A1) depicted that the lipid vesicles in TBA/water 1:4 system possessed a double layer structure (confirmed by the TEM micrographs, Figure 2) whose inner and outer aqueous phase encapsulated sCT simultaneously. It was reported by de Waard et al. that the particle size of crystal nucleus would be negatively proportion to the freezing rate (de Waard et al., 2008). Because snap freezing provided a considerable fast freezing rate in present study, it was reasonable to infer that both the inner and outer aqueous phase rapidly solidified into crystals as small as nano-scale. The hydrophilic head groups of lecithin then aggregated and assembled on the surface of these crystals (A2 of Figure 6). After freeze-drying, the TBA/water co-solvent was removed. The whole system thus become anhydrous and only nano-scale sCT core remained in the inner and outer aqueous phases. With the addition of ethanol absolute, the hydrophilic head groups of lecithin absorbed to the surface of nano-scale sCT core, while the hydrophobic chains freely stretched in the organic phase. The ARM-NPs system was formed, by which sCT was homogenously dispersed in the organic phase (A3 of Figure 6). Since the nano-scale sCT was actively encapsulated by lecithin, the obtained ARM-NPs also possessed uniform nano-metered particle size. The steric barrier effect offered by the reverse micelle vesicles and homogenous particle size made it possible to overcome the sedimentation instability during storage.

As for the TBA/water 3:2 system, both the lecithin and the sCT were originally freely dissolved in the single solution (B1 of Figure 6). During snap freezing, nano-scale of crystal of TBA hydrate instead of ice generated. The hydrophobic chains assembled in the core of the TBA hydrate crystal while the hydrophilic head groups faced toward the unfrozen outer aqueous phase, constructing a solid micelle nanostructure. Here, sCT was still dissolved freely in the bulk aqueous phase without neither being encapsulated in the nano-scale lecithin micelle, nor forming nano-scale crystal nucleuses (B2 of Figure 6). When freeze-dried, the solvent was sublimated and sCT crystal nucleuses with various sizes formed. Thereafter, this resulted in passive encapsulation of sCT and inhomogeneous size (probably not nano-scale) was obtained when ethanol absolute was added (B3 of Figure 6). Inhomogeneous size would finally cause the enlargement of the particle size and consequently sedimentation instability in pMDI, owing to the unavoidable Ostwald ripening phenomenon (Welin-Berger & Bergenstahl, 2000). It could be foreseen that such system would not be stable even though reverse micelle vesicles also produced steric barrier effect here.

This hypothesis was further confirmed by the DSC analysis. TBA/water 1:4 system underwent an l.s. – (ice + l.s.) – (ice + eutectic A) frozen crystallization behavior, while TBA/water 3:2 system successively went through l.s. – (TBA hydrate + l.s.) – (TBA hydrate + eutectic A) phases, as shown by the DSC results. The different frozen crystallization behaviors exerted a significant influence on the formation process of the ARM-NPs. Although ARM-NPs formed in both situations, their microscopic architectures were quite different. A certain frozen crystallization behavior granted ARM-NPs a capability to overcome the sedimentation instability of peptide-containing pMDI.

## Conclusion

A bottom-up process was utilized to fabricate sCT containing ARM-NPs in this study. The formulation influencing factors were investigated to determine the optimal ARM-NPs formulation. The ARM-NPs were spherical with 147.7 ± 2.0 nm particle size and 0.152 ± 0.021 PdI. The ARM-NPs based pMDI possessed a FPF of 46.99 ± 1.33%. Fourier transformation infrared and CD studies revealed that the secondary structure of sCT maintained unchanged during preparation. Stability tests demonstrated that the FPF value, content of drug per press and 1-min stability remained unchanged during the 12-week storage at 4–6 °C. The high stability of such system could be attributed to the steric barrier effect and homogenous particle size. A hypothesis was put forward to explain the formation mechanism of the ARM-NPs. The frozen crystallization behavior of different systems was then explored by DSC and confirmed the hypothesis. It was shown that the system underwent a liquid solution – (ice + liquid solution) – (ice + eutectic A) process. Taken together, the ARM-NPs were able to overcome sedimentation instability in peptide-containing pMDI.
